# Biomechanical comparison of screw vs. cerclage refixation in orthogeriatric lesser trochanteric fractures: a cadaveric study

**DOI:** 10.1007/s00068-022-02116-5

**Published:** 2022-09-27

**Authors:** Christoph Linhart, Manuel Kistler, Matthias Woiczinski, Rouven Neudeck, Matthias Kassube, Wolfgang Böcker, Christian Ehrnthaller

**Affiliations:** Department of Orthopaedics and Trauma Surgery, Musculoskeletal University Center Munich (MUM), University Hospital, LMU Munich, Marchioninistr. 15, 81377 Munich, Germany

**Keywords:** DHS, Refixation lesser trochanter, Biomechanic stability, Cerclage, Screw

## Abstract

**Purpose:**

Osteoporosis-related proximal femur fractures continue to increase significantly due to demographic change. This study was designed to evaluate the biomechanical stability of two different fixation methods (cerclage vs. screw) for refixation of a trochanter minor fragment in the pertrochanteric fractures in cadaveric bones.

**Methods:**

Artificial bones (*n* = 14) and human bones (*n* = 16) were treated with a DHS and the trochanter minor fragment was reduced by cerclage wiring or direct screw fixation. After preloading the simulated iliopsoas with 10 N, a tensile test was performed, ending with either a 70% loss of strength or avulsion of the fragment. The mean values of the avulsion force and the surface strain were recorded.

**Results:**

All tensile tests showed no significant differences between refixation using a direct screw or wire cerclage, for both artificial bones and human specimens. Absolute values showed higher avulsion forces after direct screw fixation than refixation with a wire cerclage. The surface tension of specimens treated with direct screw fixation was lower than that of specimens treated with wire cerclage. An opposite effect was seen in artificial bones. Both effects were not statistically significant.

**Conclusion:**

Based on the equal stability after lag screw placement compared to cerclage wiring, we promote the placement of a lag screw into the lesser trochanter fragment in pertrochanteric femur fractures when using a dynamic hip screw.

**Level of evidence:**

Level III.

## Introduction

Fractures of the proximal femur are among the most frequent injuries in orthopedic trauma surgery. Those fractures often occur during low-energy-trauma, such as a tripping fall, which is common for elderly people [1, 2]. The ongoing demographic change will furthermore increase proximal femur fractures among elderly patients [3]. In Germany, the incidence for proximal femur fractures is 90/100.000 inhabitants [4, 5]. Around 100.000 patients are affected every year, including a perioperative mortality rate within the first year of 5.7 up to 20% [6–8]. As a consequence, this would lead to double the amount of cases with femur fractures by 2040 [9]. According to calculations, the annual number of patients with proximal femur fractures will increase up to 6.3 million per year [10, 11].

In AO type 31.A1.3 as well as A2 proximal femur fractures according to AO (Arbeitsgemeinschaft für Osteosynthesefragen), the lesser trochanter fractured. Furthermore, fractures on the lesser trochanter can also occur isolated (AO type 31.A1.1o) or during periprosthetic fractures (Vancouver AL). If the lesser trochanter is affected during proximal femur fractures, the fracture is generally assumed to have a lack of medial support and therefore instability [12]. Previous analyses have shown that the medial cortex is significantly more important than the lateral cortex when referring to the axial stability of the proximal femur [13]. Furthermore, the iliopsoas muscle, the most important flexor of the hip, attaches to the lesser trochanter with a very high tensile force. Some authors recommended refixation of the lesser trochanter, due to a potential dislocation and consecutive decreased hip flexor strength [13, 14]. Nevertheless, refixation of the lesser trochanter fragment is usually neglected or remains at the preference of the surgeon [15–18].

Surgical treatment of proximal femur fractures is usually performed using either a dynamic hip screw (DHS, DePuySynthes® Inc., Oberdorf, Switzerland) or a proximal femoral nail with a hip component. The current research data does not prove clearly which of the two procedures offers a better clinical outcome [19–21], whereas a tendency toward the use of cephalomedullary nails can be observed.

So far in case of intraoperative refixation of the lesser trochanteric fragment, the standard procedure is open reduction and implantation of a wire cerclage around the lesser trochanter. However, this procedure causes an extended time of surgery and results in increased blood loss and soft tissue damage, which combined lead to an increased surgical risk for the patient [22, 23]. A retrograde fixation of the lesser trochanter using a Suture Button© was only used in adolescents in a few cases and is not a standard surgical procedure so far [24]. Although intramedullary implants are on the rise for treating of these fractures, globally, a dynamic hip screw represents the most widespread implant. One great advantage of the DHS could be the possibility to put a lag screw into the lesser trochanter fragment using an existing screw hole.

The aim of this study is to provide the biomechanical basis for decision-making on the optimal choice of the type of refixation of the lesser trochanter which is performed after implantation of a dynamic hip screw (DHS) once by means of a wire cerclage and once by means of a screw refixation of the lesser trochanter by orthogeriatric patients.

## Methods

### Instrumentation

Our study included two biomechanical tests. The initial testing started with artificial bones, while the final testing was performed on a human cadaver. After obtaining approval from our institutional review board, a total of 14 biomechanical artificial bones (Sawbones Europe AB, Malmoe, Sweden, Femur, PCF 17, Medium, 4th Generation Composite) were used for the first part of this study. They were split up in two groups, each containing seven artificial bones. For the second part, 16 fresh frozen human femora were commercially obtained from Science Care (Phoenix, AZ, USA). The 16 human femora were split up into two groups, each consisting of 8 specimens. The femora were frozen at − 24 °C and thawed at room temperature 24 h before experimental testing. To increase comparability and reduce bias, only matched pairs of femora were used.

The surgical technique of DHS implementation was performed according to the manufacturer´s instructions (DePuySynthes, Oberdorf, Switzerland). First, anteversion of the femoral neck by inserting a new Kirschner wire anterior to the femoral neck was determined. Then, a new DHS guide wire with the appropriate aiming device at the desired angle was inserted. The guide wire should be placed at the center of the femoral head and extend into the subchondral bone. After radiological confirmation of the correct guide wire position, the length of the DHS blade was measured with the measuring rod directly on the guide wire. When the guide wire was inserted into the subchondral bone, 10 mm from the reading was substracted. The three-step method was used to drill the length of the selected implant. Under fluoroscopy, the guide wire was checked for any migration during drilling. Then, the desired DHS plate and DHS screw were inserted over the guide wire to the desired depth. Using the centering sleeve, the DHS plate was placed flush onto the bone. 4.5 mm self-tapping cortical screws were used to fix the plate onto the bone.

**Fig. 1:**
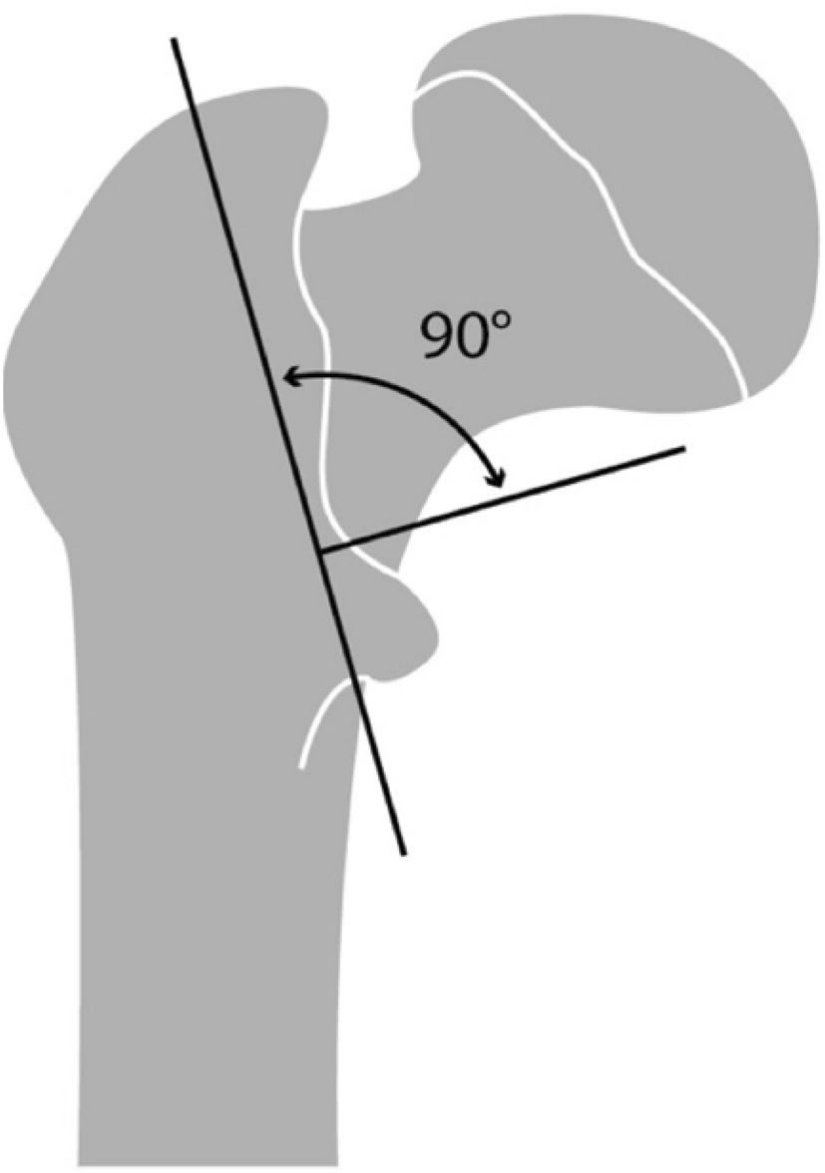
Graphic representation of the osteotomy planes for creating the lesser trochanter fragment

**Fig. 2:**
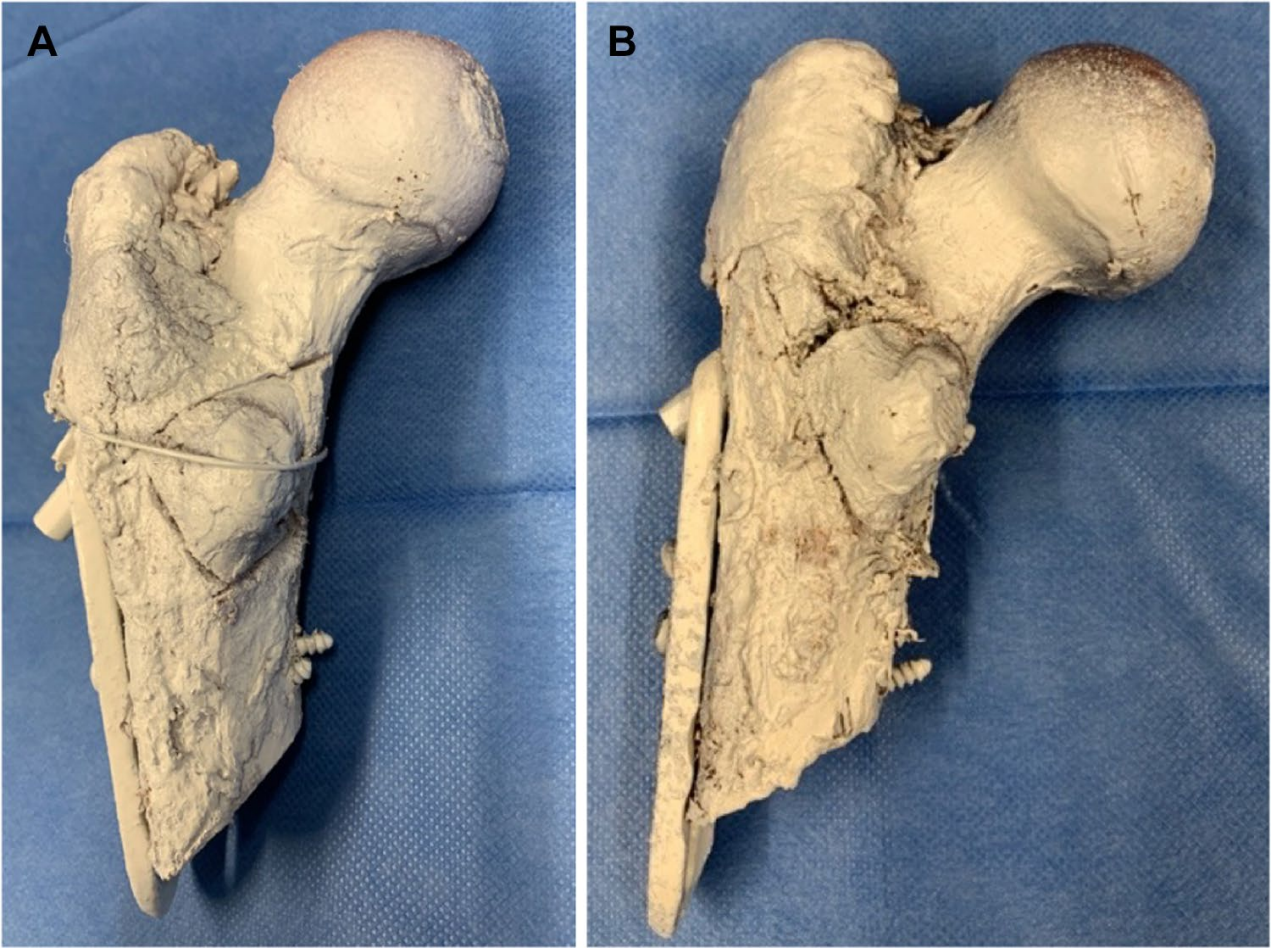
Human proximal femur with white pattern set for the optical measuring system treated with a DHS and refixed trochanter minor fragment: **A** with a cerclage. **B** with a screw

The osteotomy of the lesser trochanter was already described elsewhere [22] and is shown in Fig. 1. Shortly, the osteotomy was set standardized with a handsaw, beginning lateral of the greater trochanter tip moving to the medial cortex, right below the lower trochanter. A second osteotomy was made starting above the lesser trochanter running distally to the first osteotomy meeting the lateral perpendicularly. Depending on experimental group allocation, refixation of the lesser trochanter was either performed by placing a cerclage wire (Fig. 2A) over the top of the fragment with the wire adequately tensioned [22] or a lag screw (Fig. 2B) was placed from the first hole of the DHS plate toward the peak of the lesser trochanter fragment. At last, a horizontal hole was drilled through the lesser trochanter fragment from the medial to the lateral side to guide the steel rope.

### Biomechanical testing

**Fig. 3:**
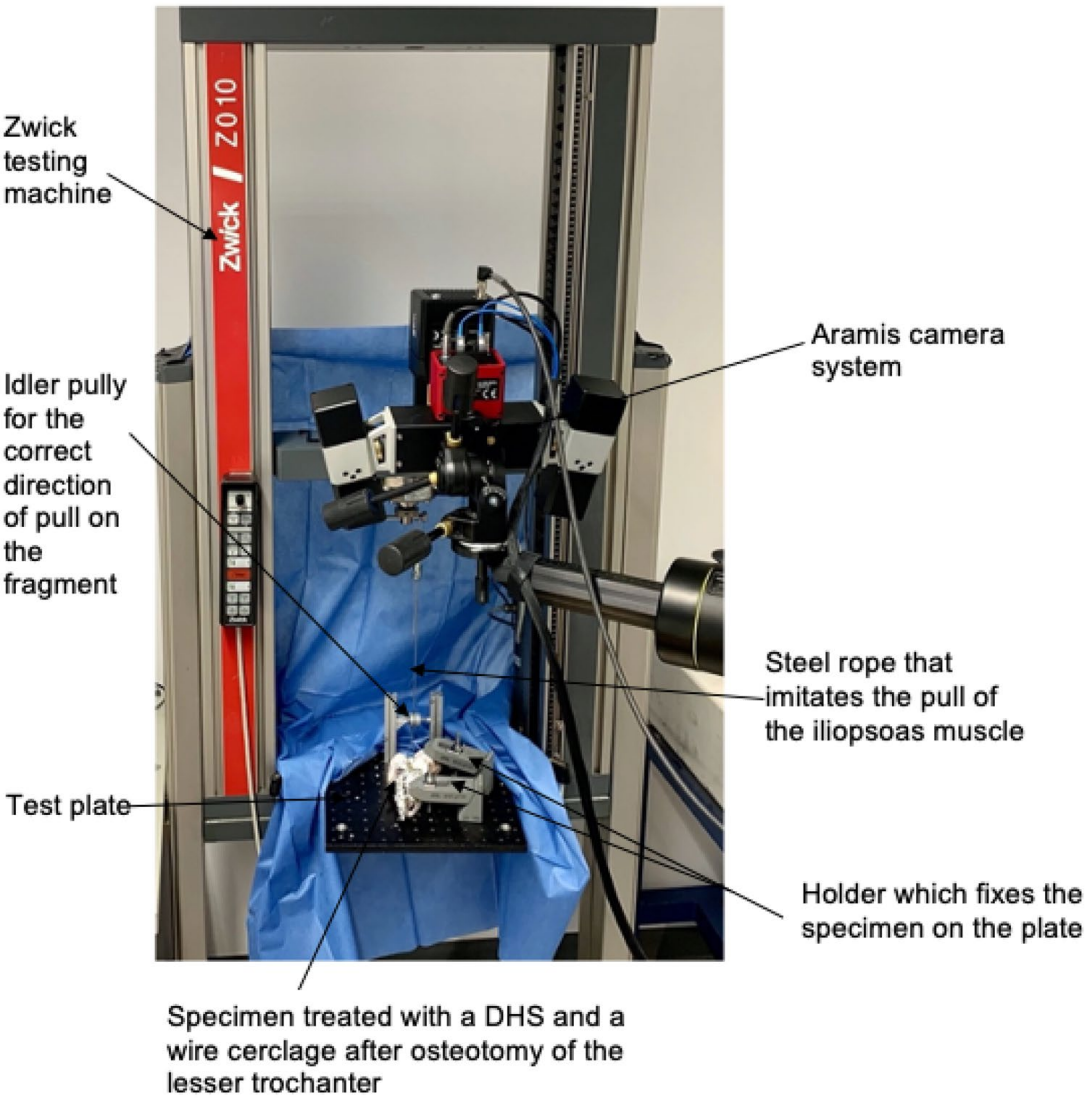
Test setup with fixed proximal femur on the base plate in the Zwick testing machine with optical measuring system. The simulated muscle is loaded via a guide roll

**Fig. 4:**
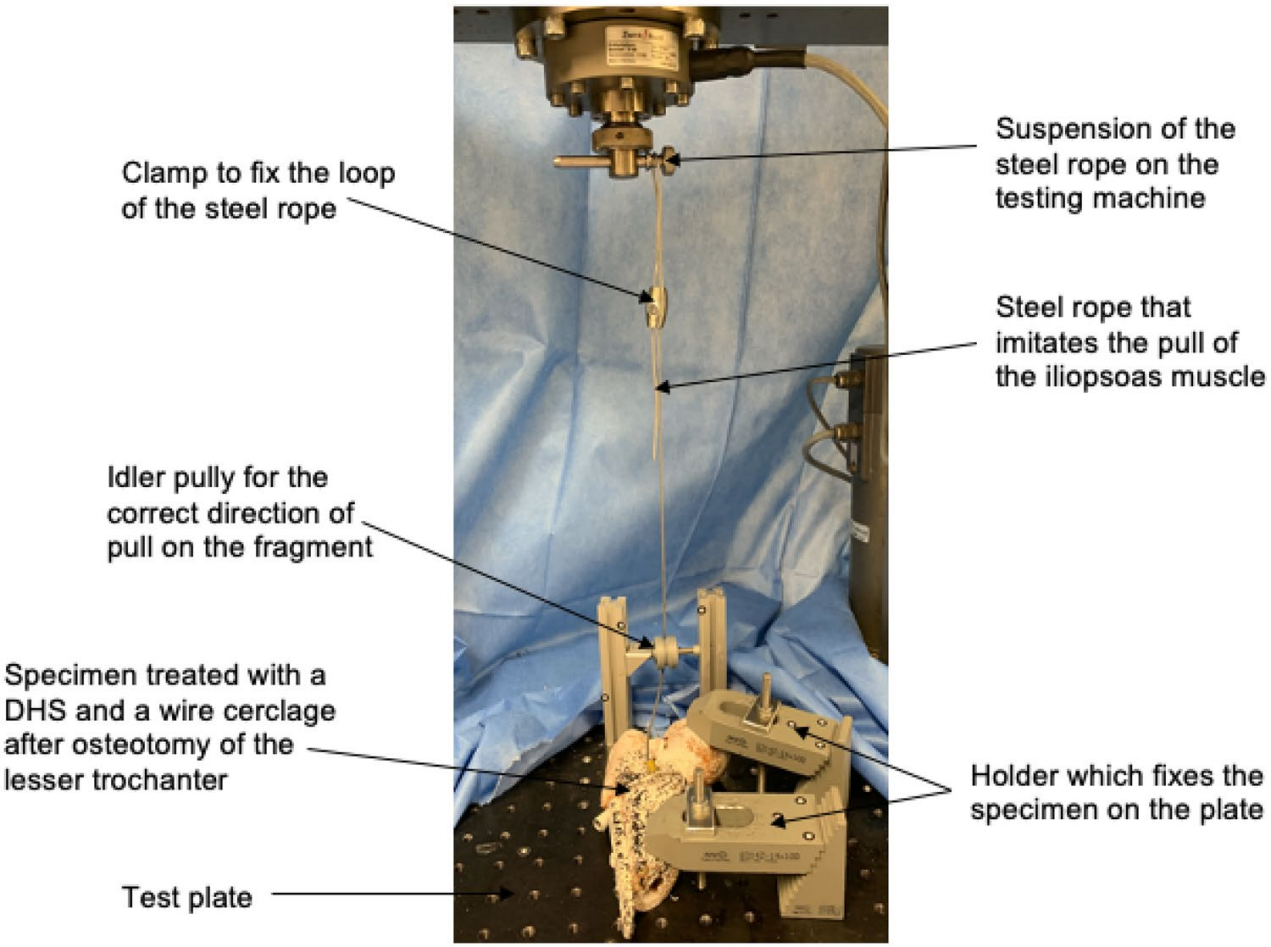
Biomechanical test setup with clamped proximal femur on baseplate and simulated iliopsoas muscle in the testing machine

**Fig. 5:**
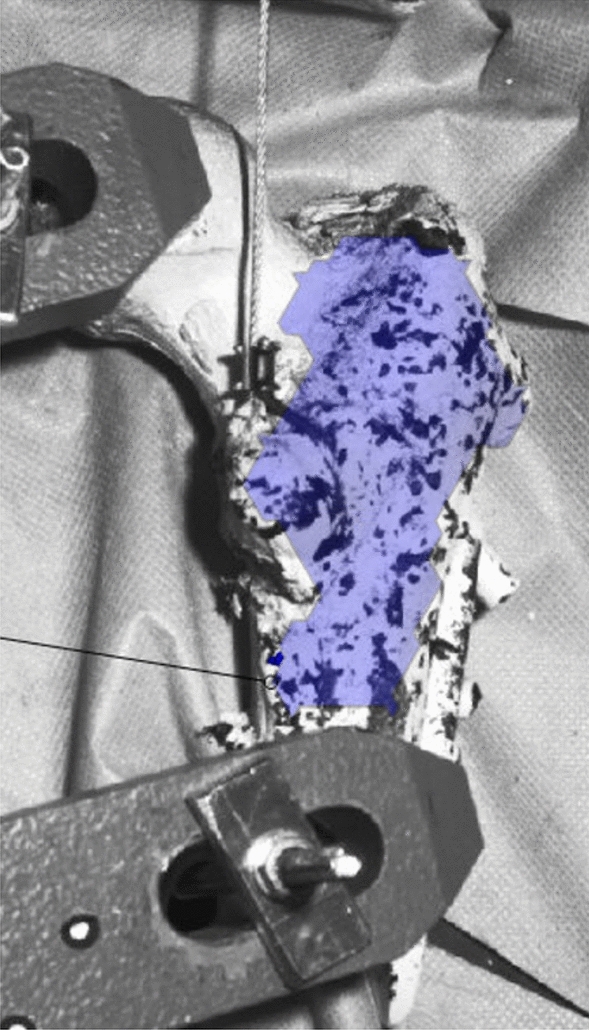
Detected surface component (blue) for measuring the surface strain with the camera system

The specimen was mounted in a material testing machine (Zwick Z10, Zwick GmbH & Co. KG, Ulm, Deutschland) and filmed with a camera system with a resolution of 1936 × 1216 pixel (ARAMIS 3D Camera 2.3 M, GOM GmbH, Braunschweig, Deutschland) (Fig. 3). This camera system is used for surface inspection and component changes in various industries. Due to the large number of images and the high resolution, it allows a detailed evaluation of changes in the surface of the specimen and a statement about any weak points. To ensure proper recognition of the surface by the camera system, specimens were colored with a black–white dot pattern. The camera system was mounted on a tripod in front of the material testing machine with a direct view upon the specimen. The specimen was mounted on a horizontal table and fixed at two points. For this purpose, two clamps were used, which could be steplessly adjusted in height and a contact pressure was applies to the specimen via a screw. One fixation point was realized over the femoral head and the second at the distal end of the fragment. A 3 mm steel rope simulating the pull of the iliopsoas muscle on the lesser trochanter fragment (Fig. 4) was used. After the iliopsoas had been preloaded with 10 N, the testing machine started to pull the fragment in the direction of the iliopsoas muscle origin, with a speed of 10 mm/min. The tests stopped after a 70% loss of maximum force or if the fragment was torn out. The camera system started the video recording simultaneously after 10 N preload with five frames per second (5 Hz) and was switched off as soon as the testing machine stopped. Biomechanical stability was assessed by measurement of the largest pull-out load (N) of the fragment before force loss of 70%. Additionally, the colored dot pattern was used to track the amount of the surface strain (in %). The surface area was determined by the pattern on the specimen and included the surface below the larger trochanter through the area of the lesser trochanter to the proximal part of the femoral shaft. By using the software GOM Correlate (GOM GmbH, Braunschweig, Deutschland), the surface component was created (Fig. 5) and the average surface strain of this area was measured.

After the biomechanical testing, the data were analyzed using the testXpert software (Zwick, Ulm, Germany), GOM Correlate (GOM GmbH, Braunschweig, Deutschland), SPSS (IBM Corp. Released 2020. IBM SPSS Statistics for Macintosh, Version 27.0. Armon,NY: IBM Corp) and Excel (Microsoft, Released 2021, Microsoft Excel for Macintosh, Version 16.50).

### Statistical analysis

For data collection and analysis, SPSS (IBM Corp. Released 2020. IBM SPSS Statistics for Macintosh, Version 27.0. Armon,NY: IBM Corp) and Excel (Microsoft, Released 2021, Microsoft Excel for Macintosh, Version 16.50) were used. All charts were created with Prism 9 (GraphPad Software Inc., San Diego, USA). The Shapiro–Wilk test was used to evaluate the data for normal distribution. Homogeneity of variances was evaluated using the Levene test. For homogenous variances, a *t* test for unpaired samples was used. For non-homogeneous variances, the Welch test was used. Differences with *p* ≤ 0.05 were considered statistically significant.

## Results

### Tensile test and surface strain in artificial bones

**Fig. 6:**
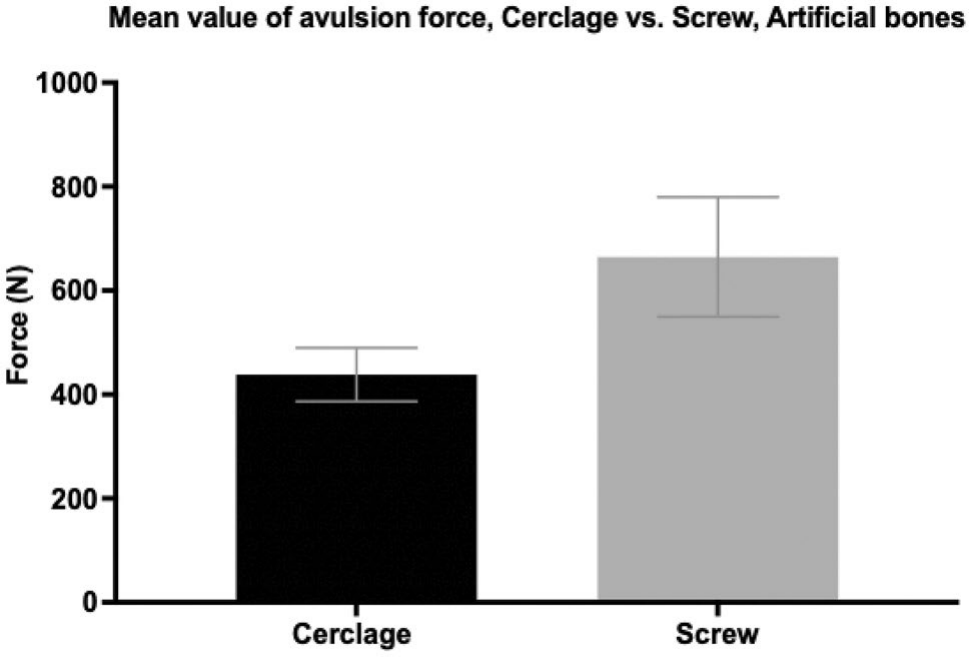
Tension forces (in newton) needed for avulsion of the lesser trochanter fragment in pertrochanteric femur fractures after DHS implantation and refixation of the lesser trochanter fragment by either cerclage wire or screw osteosynthesis. Data expressed as mean ± 2SEM (standard error of the mean)

The average force needed to cause avulsion of the lesser trochanter fragment in artificial bones treated with screw osteosynthesis was higher (664.61 N ± 115.00 N) than in specimens treated with a wire cerclage (438.24 N ± 51.45 N) (Fig. 6). Although failing to show statistical significance (*p* = 0.069), the mean average tension force for screw osteosynthesis was 34% higher compared to wire cerclage.

**Fig. 7:**
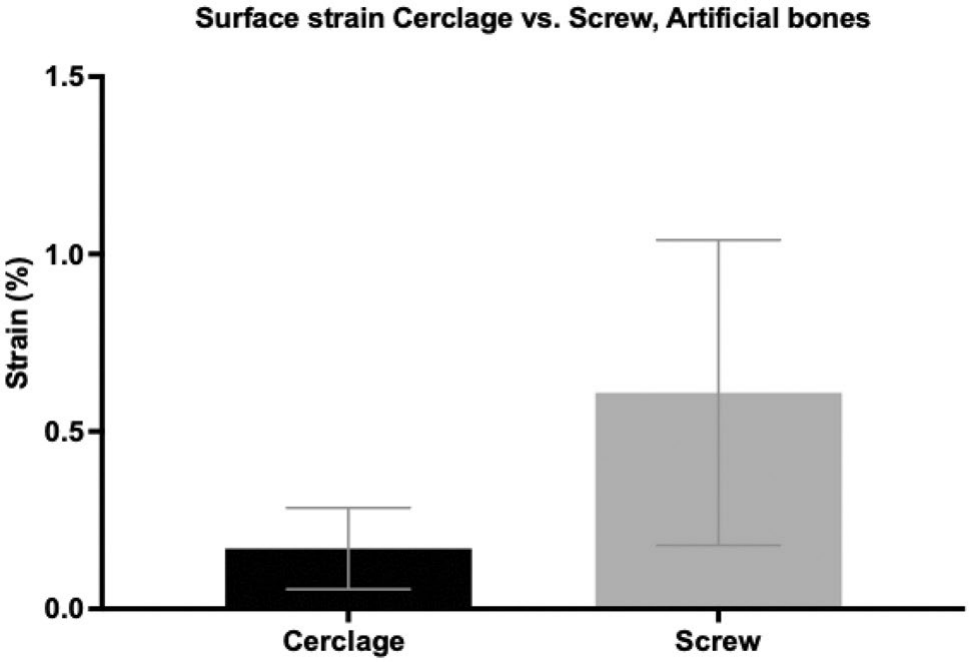
Surface strain (in %) in pertrochanteric femur fractures after DHS implantation and refixation of the lesser trochanter fragment by either cerclage wire or screw osteosynthesis. Data expressed as mean ± 2SEM (standard error of the mean)

Correspondingly, the average surface strain was lower in specimens using a wire cerclage (0.17% $\pm$ 0.11%) than in femora after screw osteosynthesis (0.61%$\pm$ 0.43%) (Fig. 7). Statistical analysis showed no significant difference (*p* = 0.344).

### Tensile test and surface strain in human femora

**Fig. 8:**
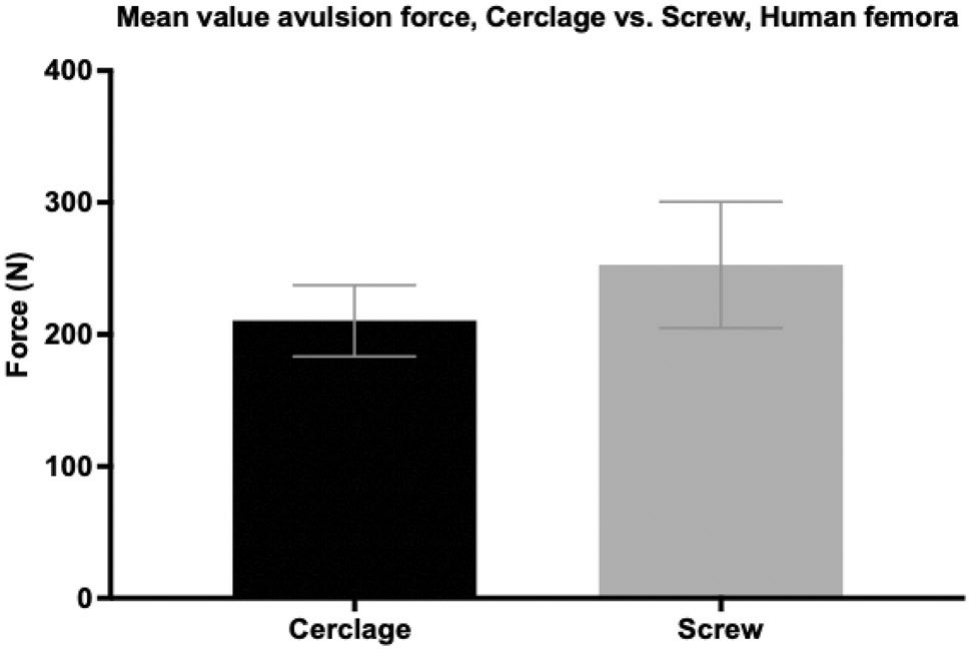
Tension forces (in newton) needed for avulsion of the lesser trochanter fragment in pertrochanteric femur fractures after DHS implantation and refixation of the lesser trochanter fragment by either cerclage wire or screw osteosynthesis. Data expressed as mean ± 2SEM (standard error of the mean)

**Fig. 9:**
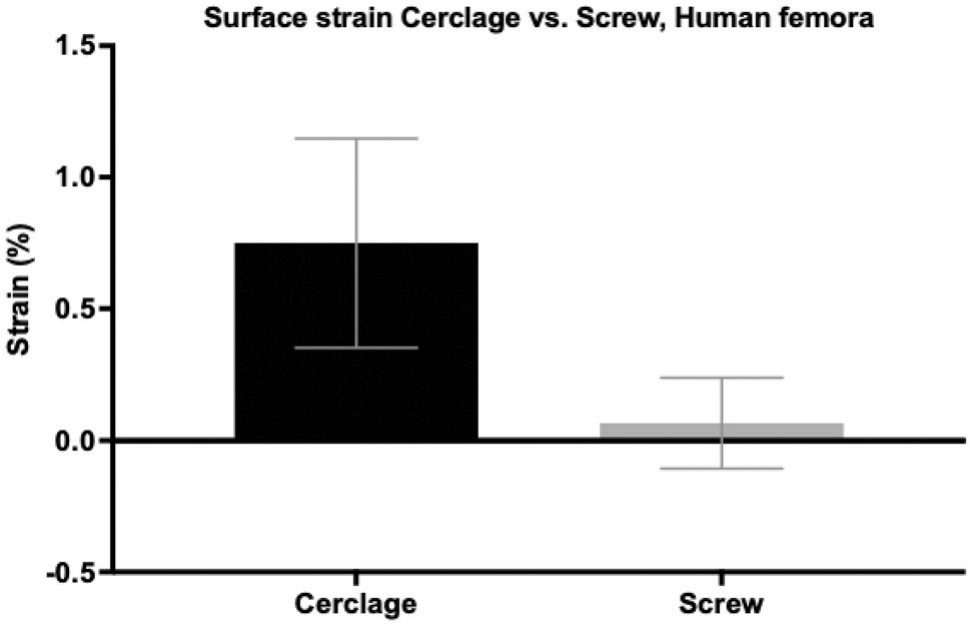
Surface strain (in %) in pertrochanteric femur fractures after DHS implantation and refixation of the lesser trochanter fragment by either cerclage wire or screw osteosynthesis. Data expressed as mean ± 2SEM (standard error of the mean)

Similarly to the tensile test with artificial specimen, the average force needed to cause avulsion of the lesser trochanter fragment in human specimen after screw osteosynthesis was higher (252.60 N $\pm$ 47.93 N) than in human femora treated with a wire cerclage (210.28 N $\pm$ 26.83 N) (Fig. 8). Although screw osteosynthesis showed 17% higher tension forces needed for avulsion of the lesser trochanter fragment compared to wire cerclage osteosynthesis, statistical analysis showed no significant difference (*p* = 0.454). The average surface strain around the fragment was higher in the group treated with wire cerclage (0.75% $\pm$ 0.40%) than in femora after screw osteosynthesis (0.07% $\pm$ 0.17%) (Fig. 9). Statistical analysis showed no significant difference (*p* = 0.137) between both groups.

## Discussion

This study was designed to evaluate the biomechanical stability of two different fixation methods for refixation of a trochanter minor fragment in pertrochanteric fractures in the orthogeriatric population. Therefore, a biomechanical pull-out test with gradually increasing loads was performed. Loss of tension as well as dislocation of the trochanter fragment was recorded. We were able to provide the first biomechanical analysis of different fixation methods for refixation of the lesser trochanter in pertrochanteric femur fractures. It was shown that a lag screw placement through the plate hole of the dynamic hip screw achieved an equivalent stabilization of the trochanteric fragment than the use of a cerclage wire. Although it is generally accepted that a posteromedial instability can have significant influence on the stability after osteosynthesis [15–18], refixation of the lesser trochanter is not generally advised until now.

The importance of the lesser trochanter fragment for the stability of the proximal femur was already highlighted by finite-element studies showing that stress upon the implant surface increases with medial instability [25] and by another study showing a gradual decrease of stability with increasing size of a lesser trochanter fragment [16].

Initial biomechanical studies were able to demonstrate increased primary stability after cerclage wiring of the lesser trochanter fragment in osteoporotic pertrochanteric fractures after intramedullary osteosynthesis [22]. Consecutive studies showed similar results regarding stability with a modified candy-package cerclage technique using two cerclage wires placed above and below the apex of the lesser trochanter [26].

The results of latter studies clearly correlate with findings of the aforementioned finite element studies supporting the theory of a beneficial role for refixation of the LT fragment.

Besides missing of a definite proof for beneficial effects of a surgical lesser trochanter refixation in clinical application, first clinical indicators were mentioned by Chang et al. examining the radiographical medial cortical support (LT fragment reduced) after fixation of pertrochanteric fractures. He was able to demonstrate that patients with reduction of medial cortical integrity had the least loss in neck–shaft angle and neck length, were walking much earlier, and displayed good functional outcomes and less hip–thigh pain presence than patients with negative reduction [27].

These results were confirmed by studies demonstrating a correlation of hip flexion strength and dislocation of the lesser trochanter [14] as well as reports about a symptomatic compression of local nerves and vessels by a dislocated trochanter minor fragment [28].

Controversial data were published by Liu et al. in a retrospective analysis of 85 intertrochanteric, intramedullary stabilized fractures [29]. Regarding postoperative complications and functional outcome, the authors were not able to find significant differences in patients with or without an additional dislocated lesser trochanter fragment. Limitations of the study should be seen in the unstandardized size of the LT fragment as well as the health status of the patient collective (62% ASA I/ II) with no evidence of an underlying osteoporosis. As it is known, that the size of the LT fragment significantly alters the mechanical stability [16] and mechanical instability might be compensated in young and healthy patients due to absence of osteoporosis, these results should be interpreted with caution.

Discussing a possible benefit for the lesser trochanter fragment with orthopedic trauma surgeons always raises doubts and questions about the relationship for the additional surgical trauma involved in this procedure possibly outweighing the biomechanical benefits of a refixation. Possible complications of a surgical lesser trochanter refixation, such as increased risk of bleeding, infections and incarceration of vessels/ nerves, have to be taken into account when considering this kind of surgical technique.

While it must be admitted that surgical incision as well as dissection and consecutively risk for complications will increase in cases with intramedullary nail osteosynthesis, no need for additional dissection is necessary when using a dynamic hip screw. Therefore, the risk for possible complications should be significantly decreased as well. The dynamic hip screw has the additional possible benefit of potentially using a screw hole opposite to the lesser trochanter fragment for placement of a lag screw for refixation of the lesser trochanter.

Although nowadays many surgeons prefer intramedullary devices due to its minimally invasive, time-sparing approach as well as theoretically better biomechanical intramedullary load distribution [21], the dynamic hip screw globally is still the most used implant for the treatment of proximal femur fractures. Until now, no superiority of one implant over the other has been clearly demonstrated [30].

Therefore, increase of care quality in this collective might show great effects in respect to patient care and socio-economic impact.

If we now compare our result of equivalent stability after lag screw osteosynthesis with that of cerclage wires with reduced risk profile for refixation of the lesser trochanter when using the dynamic hip screw, then this should be the reason to reconsider one's own surgical procedure.

Possible limitations of the study arise from the clinical transferability of our biomechanical results. Even after successful refixation of the lesser trochanter, traction of the iliopsoas muscle would repeatedly be applied at the lesser trochanter fragment, which could ultimately lead to dislocation of the fragment after loosening of the cerclage wire. Additionally, repeated gait cycles could also result in loosening of the cerclage wires with the positive effect of lesser trochanter refixation only being transient. The fact that an explicit DXA measurement of the bone donors is missing is a possible limitation, but the donors were selected according to orthogeriatric criteria (female, over 75 years old, only matched pairs used). These criteria best reflect the orthogeriatric reality and daily routine in emergency departments. The strengths of the study are the reproducible fracture patterns and the standardized measurement of the dislocation. After the preliminary tests on artificial bones, this study was carried out exclusively on human femora, which represents a much more realistic test setup than numerous published studies that were only carried out on artificial bones.

## Conclusion

Based on the equal stability after lag screw placement compared to cerclage wiring, we promote the placement of a lag screw into the lesser trochanter fragment in pertrochanteric femur fractures when using a dynamic hip screw in the orthogeriatric population. By application of this technique, the surgeon combines the biomechanical advantages of lesser trochanter refixation with increase of primary osteosynthesis stability without disadvantages, such as additional surgical dissection and increased risk of bleeding, when using a dynamic hip screw compared to an intramedullary implant.
